# PGE_2_ Induces IL-6 in Orbital Fibroblasts through EP2 Receptors and Increased Gene Promoter Activity: Implications to Thyroid-Associated Ophthalmopathy

**DOI:** 10.1371/journal.pone.0015296

**Published:** 2010-12-23

**Authors:** Nupur Raychaudhuri, Raymond S. Douglas, Terry J. Smith

**Affiliations:** 1 Department of Ophthalmology and Visual Sciences, University of Michigan Medical School, Ann Arbor, Michigan, United States of America; 2 Department of Ophthalmology and Visual Sciences and Internal Medicine, University of Michigan Medical School, Ann Arbor, Michigan, United States of America; Cardiff University, United Kingdom

## Abstract

**Background:**

IL-6 plays an important role in the pathogenesis of Graves' disease and its orbital component, thyroid-associated ophthalmopathy (TAO). Orbital tissues become inflamed in TAO, a process in which prostanoids have been implicated. Orbital fibroblasts both generate and respond to PGE_2_, underlying the inflammatory phenotype of these cells.

**Methodology/Principal Findings:**

Using cultured orbital and dermal fibroblasts, we characterized the effects of PGE_2_ on IL-6 expression. We found that the prostanoid provokes substantially greater cytokine synthesis in orbital fibroblasts, effects that are mediated through cell-surface EP_2_ receptors and increased steady-state IL-6 mRNA levels. The pre-translational up-regulation of IL-6 results from increased gene promoter activity and can be reproduced with the PKA agonist, Sp-cAMP and blocked by interrupting the PKA pathway. PGE_2_-induced production of cAMP in orbital fibroblasts was far greater than that in dermal fibroblasts, resulting from higher levels of adenylate cyclase. PGE_2_ provokes CREB phosphorylation, increases the pCREB/CREB ratio, and initiates nuclear localization of the pCREB/CREB binding protein/p300 complex (CBP) preferentially in orbital fibroblasts. Transfection with siRNAs targeting either CREB or CBP blunts the induction of IL-6 gene expression. PGE_2_ promotes the binding of pCREB to its target DNA sequence which is substantially greater in orbital fibroblasts.

**Conclusion/Significance:**

These results identify the mechanism underlying the exaggerated induction of IL-6 in orbital fibroblasts and tie together two proinflammatory pathways involved in the pathogenesis of TAO. Moreover, they might therefore define an attractive therapeutic target for the treatment of TAO.

## Introduction

In the autoimmune thyroid syndrome, Graves' disease, the orbit becomes inflamed and undergoes extensive tissue remodeling, a condition known as thyroid-associated ophthalmopathy^2^ (TAO) [1], [2]. A cardinal feature associated with TAO is the substantial infiltration of both B and T lymphocytes within orbital connective tissues [3]–[5]. Several cytokines, including IL-6, have been implicated in the pathogenesis of autoimmune diseases [6]. Hiromatsu *et al.* [7] studied the cytokine profiles of patients with Graves' disease and TAO. They found that extra-ocular eye muscle and orbital fat from these individuals express high levels of IL-6 mRNA and that orbital volumes correlated positively with levels of these transcripts. These findings may prove particularly relevant to antibody-driven autoimmune diseases like Graves' disease since IL-6 supports B lymphocyte and plasma cell function and is a recognized cofactor in fat metabolism [8], [9].

Orbital fibroblasts exhibit a unique set of phenotypic attributes when activated by cytokines and bioactive lipids. They can generate powerful chemoattractants and proinflammatory signals. These are currently believed to underlie the susceptibility of the orbit to inflammation such as that occurring in TAO [10]. Orbital fibroblasts produce extraordinarily high levels of prostaglandin E_2_ (PGE_2_) when treated with cytokines [11]–[13]. At the heart of this response is an exaggerated induction of prostaglandin endoperoxide H synthase-2 (PGHS-2), the rate limiting, inflammatory cyclooxygenase involved in the production of PGE_2_ [14]. PGHS-2 has been found over-expressed in orbital tissues from patients with TAO [15]. Moreover, both B and T cells have substantial capacity to generate PGE_2_ through the induction of PGHS-2 which occurs in their activated states [16]–[18]. Thus, the capacity of orbital tissue in TAO to generate PGE_2_ may be increased dramatically. PGE_2_ acts on target cells through one or more EP receptors, some of which are coupled to G protein through which adenylate cyclase activation leads to increased intracellular cAMP [19].

A number of factors have been shown to regulate the expression of IL-6 in a variety of cell-types [20]–[22]. Transcriptional regulation of the IL-6 gene is complex and involves the cAMP response element (CRE)-binding protein (CREB). Phosphorylated CREB is recruited to the nucleus and complexes with CREB binding protein/p300 (CBP) [20]. The amplitude of CREB mediated transcriptional effects is determined at least in part on the nature of an interaction between CREB and CBP [23]. Specifically, the two proteins interact following phosphorylation of the Ser-133 residue on CREB. This phosphorylated protein then identifies the 94 amino acid Kix domain on CBP [24]. Thus, transcriptional up-regulation of target genes resulting from cAMP generation relies on the formation of a CREB/CBP complex.

In an earlier paper, we demonstrated that IL-1β could induce the production of IL-6 in orbital fibroblasts in an anatomically selective manner [25]. That effect was mediated through an up-regulation of IL-6 gene promoter activity and was transient, lasting for only a few hours. Since that report, several other groups have detected dramatic over-expression of PGHS-2 in orbital tissues affected with TAO, especially in the early active phase [15], [26], suggesting a state where PGE_2_ and potentially other prostanoids might be generated *in vivo*. We have described the expression and highly inducible PGHS-2 and its enzymatic partner, microsomal PGE_2_ synthase, in IL-1β, leukoregulin, and CD154-activated TAO orbital fibroblasts [11]–[13]. Here, we explore the potential relationship between PGE_2_ and IL-6 production in TAO-derived orbital fibroblasts. The induction by PGE_2_ of IL-6 is mediated through cell-surface EP2 receptors, an intermediate generation of intracellular cAMP, and the obligatory formation of a nuclear complex comprising CREB and CBP/p300. Knocking down either CREB or CBP expression dampens the level of IL-6 induction. Our findings for the first time demonstrate the potential importance of exaggerated PGE_2_ generated in orbital fibroblasts as an autocrine regulatory factor.

## Results

### PGE_2_ induced IL-6 expression in orbital fibroblasts in an anatomic site-selective, time- and dose-dependent manner

**Figure 1:**
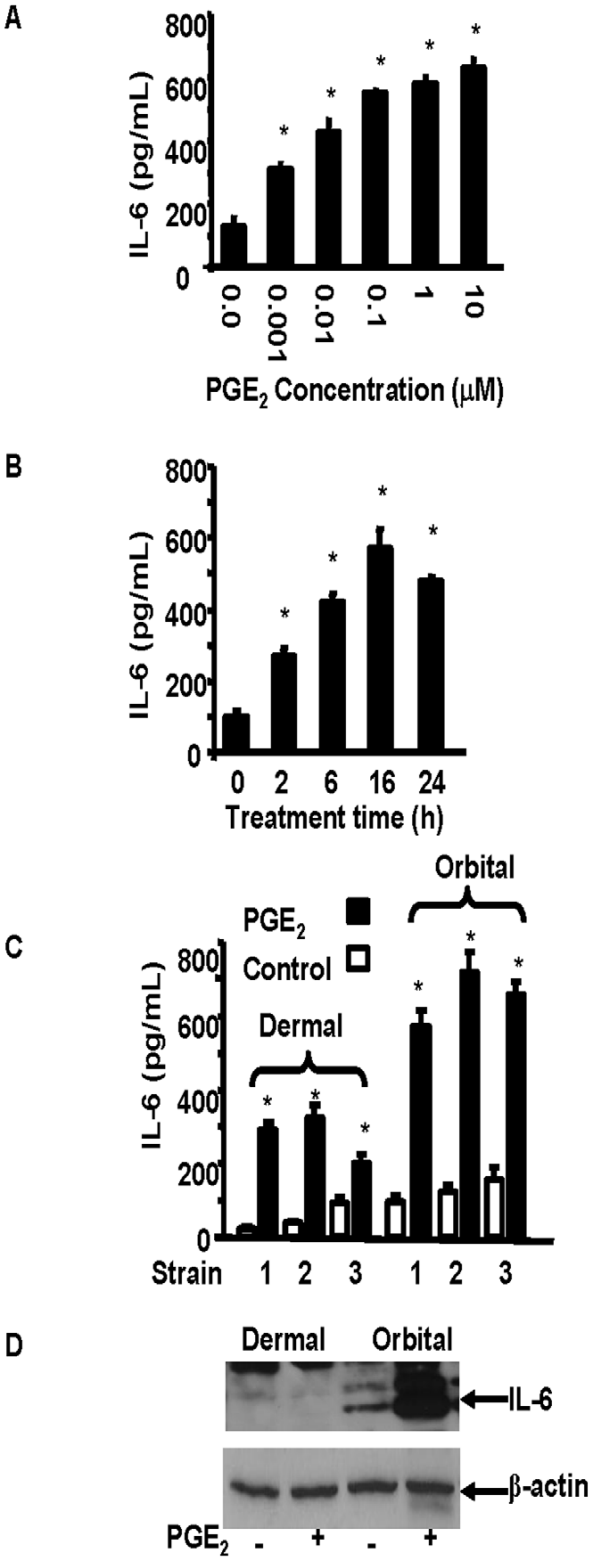
IL-6 production in orbital fibroblasts is induced by PGE_2_ in a concentration- and time-dependent manner. Confluent orbital cultures, in this case from a patient with TAO, were treated **A**: with escalating concentrations of PGE_2_ for 16 h. or **B**: with PGE_2_ (1 µM) for graded intervals or . **C**: with or without PGE_2_ (1 µM) for 16 h in three different dermal and orbital fibroblast strains, each from a different donor. Media were collected and subjected to ELISA analysis as described in *Materials and Methods*. Data are expressed as the mean ± SD of triplicate determinations. * denotes P<0.005 compared to PGE_2_ treatment alone. **D**: Orbital and dermal cultures were treated with nothing or with PGE_2_ (1 µM) for 16 h., and cell layer protein was collected and analyzed by Western blot for IL-6 protein. Membranes were then re-probed with anti-β actin antibody as a loading control. Band densities, corrected for their respective β-actin signals: Dermal control, 0.132, Dermal PGE_2_, 0.146; Orbital control, 1.098; Orbital PGE_2_, 5.132.

IL-6 production in untreated orbital fibroblasts occurred at a very low level but when PGE_2_ was added to the culture medium, levels of the cytokine were increased substantially. As the data in Fig. 1A demonstrates, even at the lowest concentration of the prostanoid tested (1 nM), an effect was detectable and reached statistical significance. The response was near maximal at 0.1 µM, where it was 4.5-fold above baseline. Increasing the concentration by 100-fold failed to increase synthesis further. The effects were rapid and at 6 hr, IL-6 concentrations in the medium had increased by nearly five-fold (Fig. 1B). They continued to increase for 16 hr when they were 5.9-fold above untreated levels (p<0.0001) and they remained elevated for 24 hr, the duration of the study. The induction was 82% greater in three orbital fibroblast strains compared to that in three from the skin (Fig. 1C, p<0.01). The level of cell layer-associated IL-6 achieved in orbital fibroblasts following PGE_2_ treatment for 16 hr. was dramatically greater than that in dermal fibroblasts (>5-fold vs <10%, Fig. 1D).

### Up-regulation of IL-6 by PGE_2_ in orbital fibroblasts involved the induction of its mRNA and gene promoter activity

**Figure 2:**
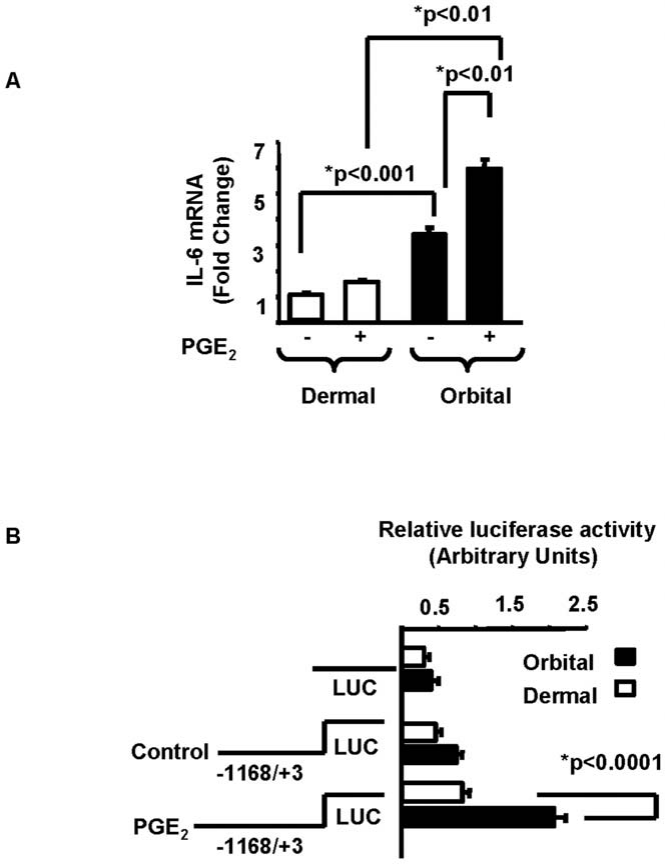
PGE_2_ upregulates IL-6 mRNA and IL-6 gene promoter activity. **A**: Divergent levels of IL-6 protein induced by PGE_2_ in orbital and dermal fibroblasts are reflected in the abundance of IL-6 mRNA. Fibroblasts were treated with nothing or PGE_2_ (1 µM) for 16 h. Ct values were normalized to GAPDH and expressed as fold-change. Data are expressed as the mean ± SD of triplicate independent determinations from one dermal and one orbital fibroblast cell. In 3 different strains of each, basal IL-6 mRNA levels were 3.2 fold greater in orbital fibroblasts. Following PGE_2_ (1 µM) for 16 h, levels were 3.7-fold higher in orbital versus dermal fibroblasts. **B**: PGE_2_ upregulated IL-6 gene promoter activity in orbital and dermal fibroblasts transiently transfected with empty luciferase vector or that construct fused to an 1171-nt fragment spanning −1168 to +3 nt of the human IL-6 gene promoter. Cultures were then treated with nothing (control) or PGE_2_ (1 µM) for 1 h. Data are expressed as the mean ± SD of triplicate independent determinations. * denotes statistical difference between groups. In another study, 3 different orbital fibroblast strains demonstrated 2.6 fold greater IL-6 promoter activity compared to dermal fibroblasts following 1 h treatment with PGE_2_ (1 µM).

The time interval between initiation of the treatment with PGE_2_ and a detectable increase in IL-6 protein suggested action at the pre-translational level. This was born out since IL-6 steady-state mRNA was increased by PGE_2_ (1 µM). As the real-time RT-PCR analysis in Fig. 2A indicates, TAO orbital fibroblasts expressed higher basal levels of IL-6 mRNA than did those from the skin. Levels in untreated orbital fibroblasts were 3.4-fold above those in dermal cultures (p<0.001 vs. dermal) and increased after 16 hr of treatment with PGE_2_. The magnitude of the increase was 1.75-fold in the orbital fibroblasts (p<0.01 vs. basal). In contrast, PGE_2_ failed to increase IL-6 mRNA levels significantly in the dermal cultures.

To determine whether PGE_2_ was acting to enhanced IL-6 gene transcription, a fragment of the IL-6 gene promoter was cloned and fused to a luciferase reporter gene, and transiently transfected into orbital and dermal fibroblasts. .After 1 hr, the reporter gene activity was significantly higher in PGE_2_-treated TAO orbital fibroblasts than that found in identically treated dermal fibroblasts (Fig. 2B). The prostanoid increased promoter activity by 2.8-fold in orbital fibroblasts while increasing the activity by only 80% in dermal cells (p<0.0001 and p<0.005, respectively).

### PGE_2_-induced IL-6 production was mediated through cAMP generation and PKA

**Figure 3:**
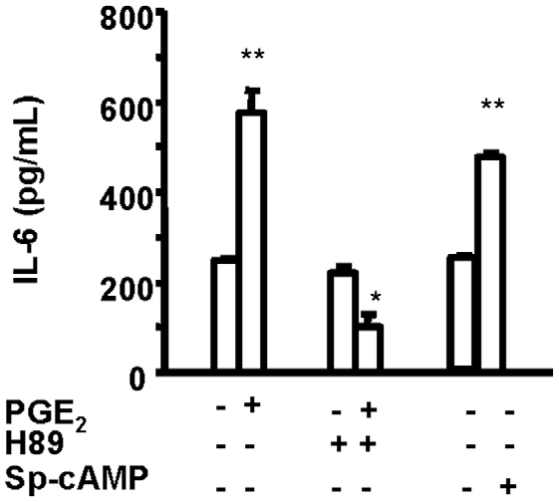
PGE_2_-induced IL-6 production can be attenuated by inhibitors of PKA. Confluent orbital fibroblasts, in this case from a patient with TAO, were treated with PGE_2_ (1 µM) or Sp-cAMP (1 mM) in the absence or presence of H89 (10 µM). Media were collected and analyzed for IL-6 content after 16 hr incubations. Data are presented as the mean ± SD of triplicate independent determinations from a single orbital fibroblast strain. . This result was confirmed in two other orbital fibroblast strains where IL-6 production was reduced 5.6 fold by H89. * denotes P<0.005 compared to PGE_2_ treatment alone; ** denotes P<0.005 compared to no treatment.

PGE2 can exert its actions through one of several signaling pathways, depending on the receptor subtype through which the cellular response was mediated. With regard to the induction of IL-6 by the prostanoid, the JAK2 inhibitor, AG490 (10 µM), PI3 kinase inhibitor, LY294002 (10 µM), JNK inhibitor, II 420119 (10 µM), and protein kinase C inhibitor, Calphostin C (100 nM) all failed to inhibit the PGE2-induced IL-6 production (data not shown). In contrast, H89 (10 µM), a specific PKA inhibitor, dramatically reduced the effects of PGE2 on IL-6 while Sp-cAMP (1 mM), a cAMP analog, mimicked PGE_2_-induced IL-6 synthesis (Fig. 3).

### PGE_2_-mediated IL-6 synthesis involved phosphorylation of CREB in fibroblasts

**Figure 4:**
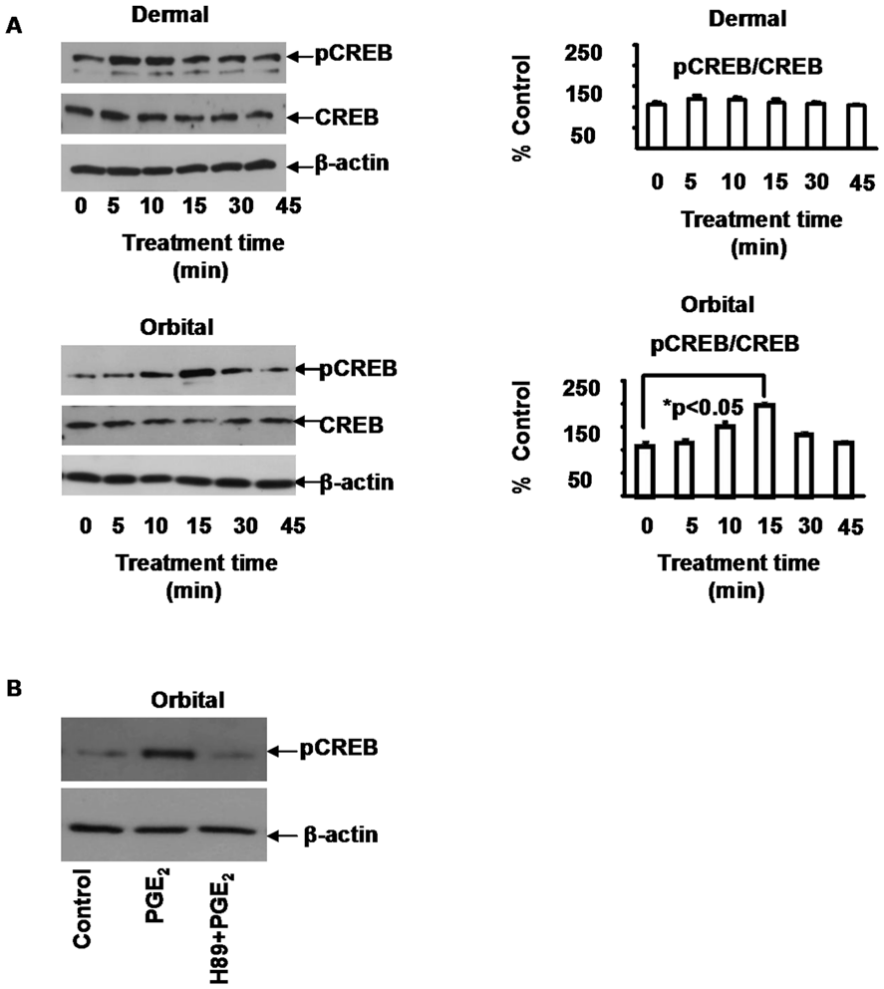
PGE_2_ provokes the phosphorylation of CREB in orbital fibroblasts, an effect blocked with H89. **A**: Confluent cultures were treated with PGE_2_ (1 µM) for different intervals. Cellular proteins were subjected to Western blot analysis of CREB and pCREB. Densitometric analysis of pCREB protein concentrations are expressed as the ratio to total CREB protein as a percent of the value at “0” min. Data are expressed as the mean ± SD of triplicate independent determinations. (* denotes statistical significance between treatment groups). **B**: H89 (10 µM) inhibits PGE_2_-provoked CREB phosphorylation in orbital fibroblasts. The inhibitor was added for 6 hrs, followed by addition of PGE_2_ (1 µM) for 15 min. Cellular proteins were subjected to Western blot analysis of pCREB protein using β-actin as a loading control.

**Figure 5:**
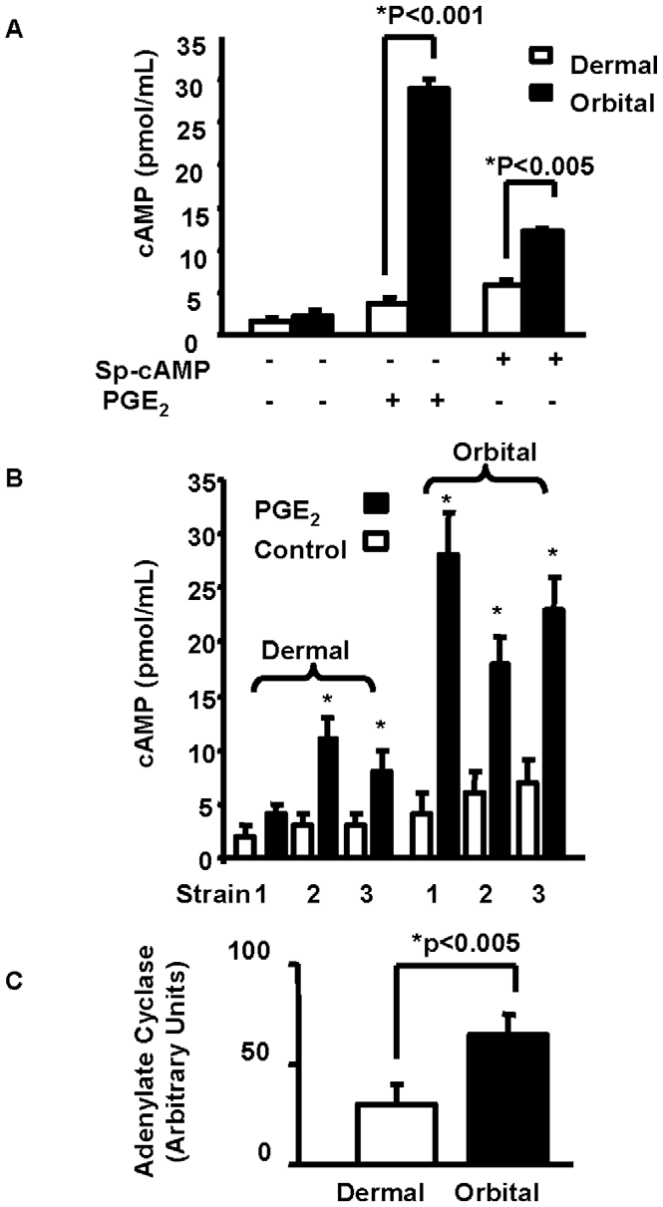
PGE_2_ induces more robust cAMP generation in orbital compared to dermal fibroblasts resulting from higher levels of adenylate cyclase. **A:** Confluent cultures were serum starved for 20 h and then treated with PGE_2_ (1 µM) for 16 hrs. Cells were then lysed with 0.1 N HCl and cAMP was measured by cAMP immunoassay. Data are presented as the mean ± SD of three independent determinations. (* indicates statistical differences between groups). **B:** cAMP levels in three different dermal and orbital fibroblast strains treated with nothing or PGE_2_ (1 µM) for 16 hrs. Data are presented as the mean ± SD of three independent determinations. The level of cAMP produced in orbital fibroblasts is 3 fold (p<0.05) greater than in dermal fibroblasts, **C:** Orbital fibroblasts express higher adenylate cyclase levels when compared to dermal fibroblasts. Data derived from Western blot analysis of three separate dermal and orbital fibroblast strains. These were normalized to their respective β-actin levels. They are expressed as the mean ± SD, p<0.005, n = 3.

PGE_2_ treatment resulted in rapid CREB phosphorylation in orbital fibroblasts, peaking at 5–15 min (Fig. 4A). The pCREB/CREB ratio remained constant in dermal fibroblasts but increased significantly in those from the orbit after 15 min of treatment. H89 abolished PGE_2_-induced CREB phosphorylation, suggesting that it was mediated through an increased intracellular cAMP concentration (Fig. 4B). To test this possibility, cAMP was measured and basal levels were comparable in untreated orbital and dermal fibroblasts (Fig. 5A). PGE_2_ (1 µM) increased cAMP generation by 12.6-fold in orbital while only 2.3-fold in dermal cells (Fig. 5A). This suggested that the orbital fibroblasts had a substantially greater capacity for generating cAMP. Generation of the cyclic nucleotide was then compared in three orbital and three dermal strains. cAMP levels were consistently higher in the orbital fibroblasts, especially following treatment with PGE_2_ (Fig. 5B). Levels of adenylate cyclase were then determined by Western blot analysis. The analysis disclosed substantially higher levels of enzyme expression in orbital fibroblasts (orbital 65±10 vs dermal 31±9 AU, p<0.005) (Fig. 5C). Thus, divergent levels of adenylate cyclase might represent the basis for the greater cAMP generation in response to PGE_2_.

### EP_2_ receptor mediated the action of PGE_2_ in TAO orbital fibroblasts

**Figure 6:**
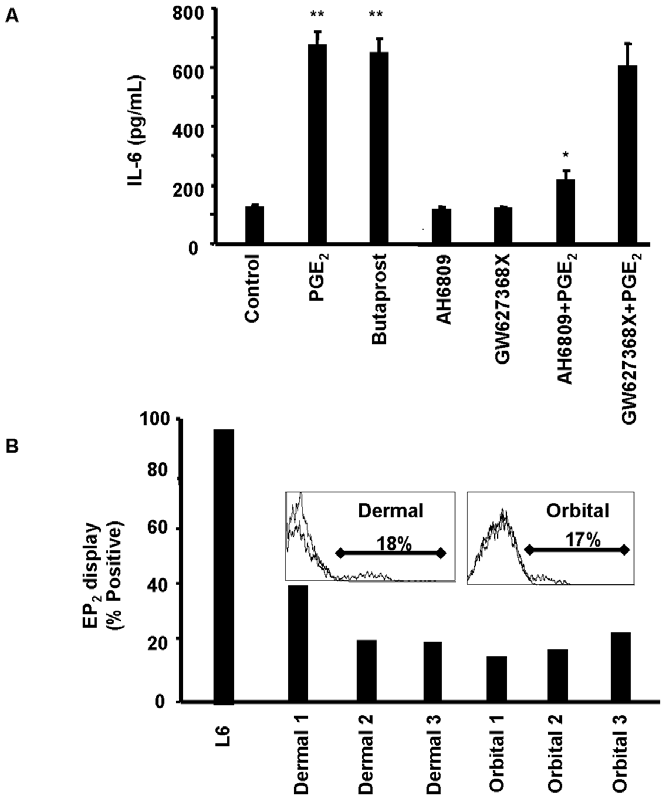
EP_2_ mediates the actions of PGE_2_ on IL-6 expression in orbital fibroblasts. **A**: Confluent cultures were treated with nothing, Butaprost (10 µM) or PGE_2_ (1 µM) in presence or absence of EP_2_ inhibitor AH6809 (10 µM), EP_4_ inhibitor GW627368X (10 µM) for 16 h. Media were subjected to IL-6 ELISA. Data are expressed as mean ± SD of three independent determinations. * denotes P<0.005 compared to PGE_2_ alone, ** denotes P<0.005 compared to no treatment. **B**: Comparison of surface EP_2_ receptor display by orbital and dermal fibroblasts as determined by flow cytometry. L6 cells serve as the positive control. Inset histograms represent the levels of shift when compared with isotype.

Several receptors displayed on the cell surface can mediate the actions of PGE_2_ [29]. Moreover, the EP_2_ subtype receptor displayed on orbital fibroblasts mediates other actions of endogenous and exogenous PGE_2_ [30], [31]. We next set out to determine whether the higher level of cAMP generation and IL-6 production in orbital fibroblasts was, at least in part, a consequence of greater EP receptor display. The EP_2_–selective agonist, Butaprost, could induce IL-6 synthesis as did authentic PGE_2_ (Fig. 6A). Dermal fibroblasts failed to respond (not shown). Moreover, the EP_2_ antagonist, AH6809 (10 µM), reduced appreciably the PGE_2_-induced IL-6 production in orbital fibroblasts. The EP_4_ antagonist GW627368X (10 µM) failed to influence PGE_2_-stimulated IL-6 production, strongly suggesting that EP_4_ is not involved in these actions of PGE_2_. We next assessed levels of the EP_2_ receptor by flow cytometry. As the flow plots shown in Fig. 6B demonstrate, the abundance of EP_2_ appeared equivalent on dermal and orbital fibroblasts despite differences in the magnitude of IL-6 induction and cAMP generation provoked by PGE_2_. Both the fraction of EP_2_^+^ cells and the receptor densities were similar in the two populations of fibroblasts. Thus, the divergent magnitude of IL-6 induction could not be attributed to differences in EP_2_ receptor levels.

### PGE_2_ promotes the formation of nuclear CREB/CBP complexes in orbital fibroblasts

Given the strong suggestion from the preceding findings, it appeared that CREB phosphorylation might play an important role in mediating the induction by PGE_2_ of IL-6.

**Figure 7:**
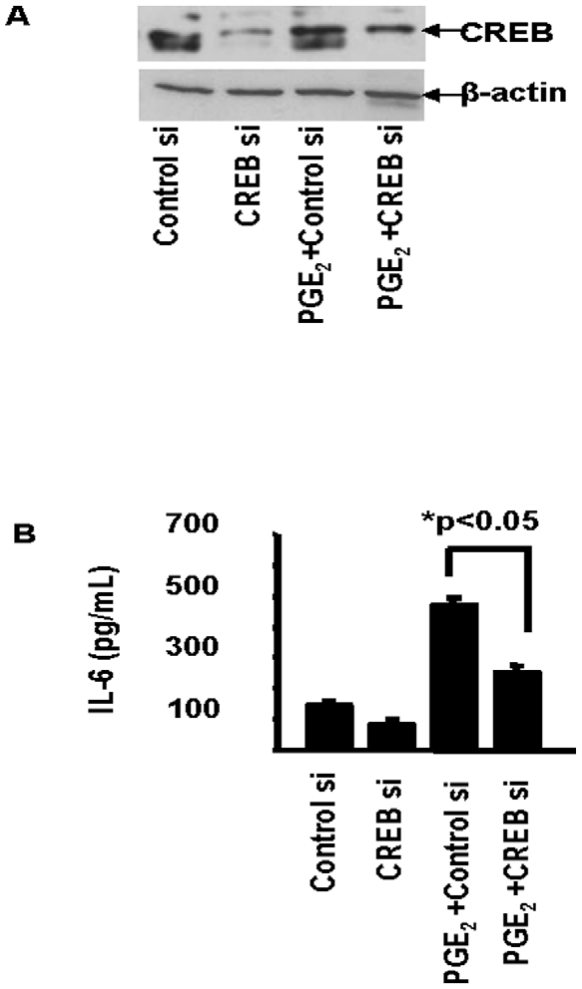
Knockdown of CREB with siRNA attenuates PGE_2_-induced IL-6 protein expression in orbital fibroblasts. **A**: siRNA specific to CREB (CREB si) or scrambled siRNA (Control si) was transfected into 80% confluent cultures. Representative Western blot analysis demonstrates the impact of the knockdown of CREB protein. **B**: Media were subjected to IL-6 ELISA. Data are expressed as the mean ± SD of three independent determinations These results were representative of those in two other orbital fibroblast strains.

**Figure 8:**
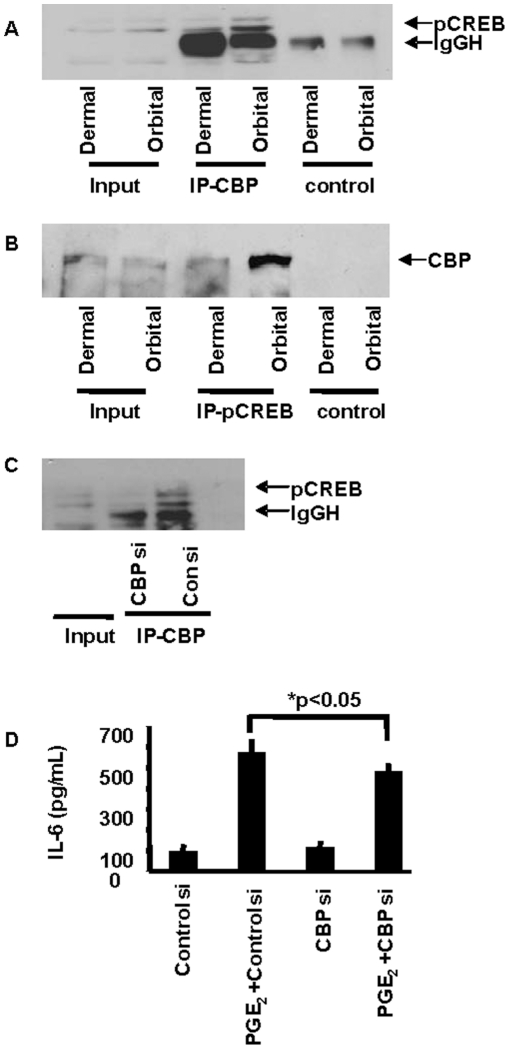
Divergent CBP/pCREB complex formation and its importance to PGE_2_-dependent IL-6 expression in orbital fibroblasts. **A**: Pull-down studies demonstrating CBP/pCREB protein-protein interactions provoked by PGE_2_. Representative Western blot demonstrates pCREB protein (arrow) in nuclear extracts from one dermal and one orbital fibroblasts (input), or following immunoprecipitation with either anti-CBP antibody (IP-CBP) or a control antibody (control). **B**: Western blot demonstrating CBP protein (arrow) in nuclear extract (input) or following immunoprecipitation with anti-pCREB antibody (IP-pCREB) or a control antibody (control). **C**: CBP knocked down by siRNA results in diminished nuclear pCREB recruitment. pCREB protein (arrow) in orbital fibroblast nuclear extracts (input) or following immunoprecipitation with anti-CBP antibody (IP-CBP) in fibroblasts transfected with CBP siRNA for 72 hr. and treated with nothing or PGE_2_ for 20 min. Note the absence of detectable pCREB in the immunoprecipitate. **D**: CBP knockdown reduced PGE_2_-induced IL-6 protein. Cultures were transfected with either CBP siRNA or control siRNA and treated with nothing or PGE_2_ (1 µM) for 16 h. Media were collected and subjected to ELISA for IL-6. Data are expressed as the mean ± SD of three independent determinations. These results were confirmed in two other orbital fibroblast strains where IL-6 production was reduced 1.2 fold by CBP siRNA.

**Figure 9:**
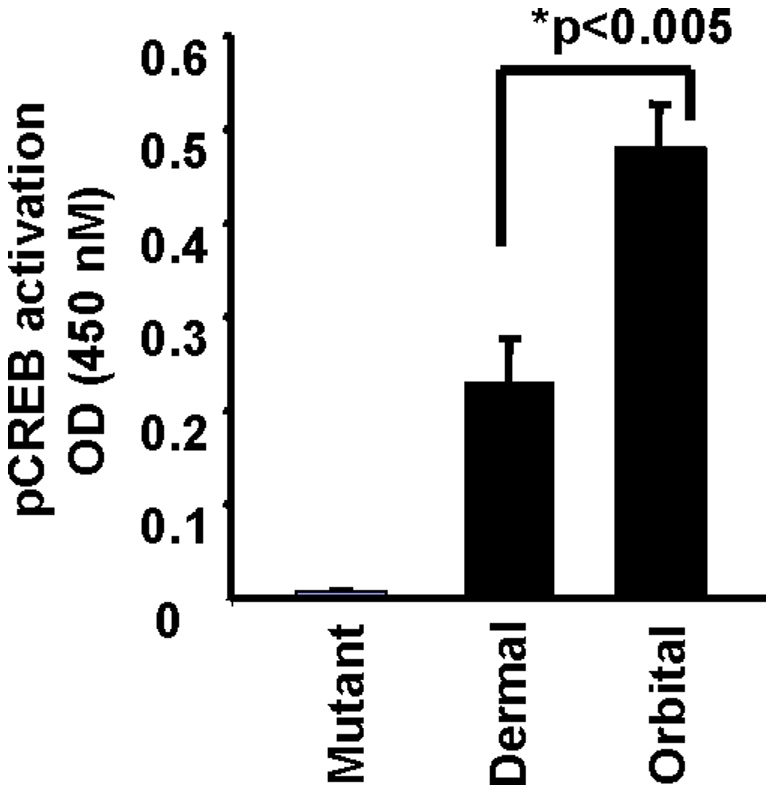
PGE_2_ enhances DNA binding of pCREB preferentially in orbital fibroblasts. Eighty percent confluent cultures were serum starved for 20 h and then treated with PGE_2_ (1 µM) for 16 h. Nuclear extracts (5 µg) were subjected to the TransAM ELISA for detecting pCREB/DNA complexes as determined at 450 nm. Data are expressed as the mean ± SD of triplicate independent determinations from a single orbital and dermal fibroblast strain. They were representative of results from 3 strains of each which demonstrated 2.1 fold greater pCREB binding in orbital versus dermal strains.

The impact of knocking down CREB with a specific siRNA on IL-6 induction in orbital fibroblasts was then determined. As Fig. 7A demonstrates, treating orbital fibroblasts with CREB siRNA could efficiently interrupt CREB protein expression, under basal and PGE_2_-treated conditions. The importance of CREB to the induction of IL-6 by PGE_2_ was then demonstrated by the attenuation of cytokine production following transfection with CREB siRNA (Fig. 7B). Optimal transcriptional activation of certain gene promoters containing CRE requires an association between pCREB and CBP [32]. We attempted to determine whether PGE_2_ influenced CREB binding to CBP. The immunoprecipitation of CBP could bring pCREB out of solution in lysates derived from orbital fibroblasts following treatment with PGE_2_ for 15–20 min. (Fig. 8A). Alternatively, subjecting these lysates to antibodies directed against pCREB resulted in immunoprecipitation of CBP complexed with pCREB from these same nuclear extracts (Fig. 8B). As the study clearly demonstrated, there was no detectable complex precipitation in nuclear lysates from similarly treated dermal fibroblasts. This result indicates that the basis for divergent IL-6 induction by PGE_2_ in orbital fibroblasts concerns, at least in part, the cell-type specific interaction between pCREB and CBP following treatment with the prostanoid. Fig. 8C demonstrates that CBP siRNA reduced the yield of pCREB co-immunoprecipitated with anti-CBP. Analogous to the findings following interruption of CREB expression (Fig. 7B), CBP siRNA could also significantly attenuate the induction of IL-6 (Fig. 8D). Thus both components of the CREB/CBP/p300 complex are necessary for an optimal induction by PGE_2_ of IL-6. PGE_2_ treatment results in a substantial enhancement of pCREB binding to target DNA (Fig. 9). The effects on DNA binding are substantially greater in orbital fibroblasts than those found in dermal cultures.

## Discussion

IL-6 exerts diverse influence on adipose tissues such as those present in the human orbit. Moreover, the cytokine has been implicated in the pathogenesis of obesity where it may determine the pattern of fat accumulation throughout the body [33]–[35]. A limited number of studies have examined PGE_2_ effects on IL-6 expression in other cell types. For instance, PGE_2_ upregulates cAMP levels in the rat intestinal epithelial cell line, IEC-6, and in so doing enhances endotoxin-induced IL-6 production [36]. In the human early leukemia T cell line, HSB.2, misoprostol, an EP_4_/EP_2_/EP_3_ selective agonist, induces IL-6 mRNA and increases IL-6 secretion, effects related to the activities of PKA but not PKC [37]. In tissue-infiltrating macrophages, the upregulation by PGE_2_ of IL-6 is mediated through EP_4_ receptors and PKC signaling [38]. None of these earlier studies examined the mechanisms involved in the transcriptional regulation of the IL-6 gene by the prostanoid.

The results presented here tie together two potentially important aspects of the divergent phenotype displayed by orbital fibroblasts. The cellular attributes peculiar to orbital fibroblasts may underlie, at least in part, their roles in the pathogenesis of TAO. In particular, they produce substantial levels of PGE_2_ when activated and can also respond to the prostanoid in a cell-specific manner [30], [31]. These responses to PGE_2_ are unusually robust and can result in dramatic morphological changes that are driven by cAMP generation. The current study sheds new insight into the mechanism underlying the high capacity for the generation of cAMP in orbital fibroblasts because we report the substantially higher levels of adenylate cyclase they express (Fig. 5B). It may have direct relevance to thyrotropin receptor function, especially in the context of Graves' disease. That receptor utilizes a G protein-coupled mechanism for cAMP generation as a principal signaling pathway in the thyroid [39]. IL-1β, leukoregulin, and CD154 promote PGE_2_ generation in these fibroblasts through a mechanism that involves the *de novo* synthesis of PGHS-2, the rate limiting synthetic enzyme in the production of PGE_2_ [12]–[14]. IL-1β and CD154 also induce IL-6 in orbital fibroblasts, unlike several other primary human fibroblasts [25], [40]. PGE_2_ exerts its own positive effects by up-regulating IL-6 gene transcription. Thus, our current findings suggest that PGE_2_ functions as a positive regulator of IL-6 production in orbital connective tissues, accounting for at least in part the high levels thought to be achieved in TAO [7].

The actions of PGE_2_ on IL-6 are mediated through the activation of EP_2_ receptors, generation of cAMP, and the recruitment of CREB/CBP/p300 complex to the orbital fibroblast nucleus. While levels of EP_2_ receptor appear similar in dermal and orbital fibroblasts, those of cAMP generated as a consequence of PGE_2_ exposure are dramatically different in the two cell types (Fig. 5A, 5B). Moreover, the magnitude of IL-6 induction was similarly divergent suggesting that the levels of cAMP generated may determine, at least in part, the magnitude of cytokine production. This appears to result from the higher levels of adenylate cyclase in orbital fibroblasts. CBP/p300 was initially implicated the transcriptional activation imposed by phosphorylated CREB [41]. It functions in a dual role within the nucleus, serving as both a histone acetyltransferase and as a transcriptional adaptor molecule [42]. The phosphorylation of CREB at Ser 133 can be mediated through the Akt pathway, results in the recruitment of CBP, and may help explain the role of CREB/CBP/p300 in enhanced cell survival [43]. CBP/p300 recruitment is mediated through changes in nuclear calcium and calcium/clamodulin-dependent protein kinase IV activity, but complex recruitment apparently does not necessarily result in CREB/CBP/p300-dependent transcriptional activation [44]. It would appear that the actions of PGE_2_ described here are mediated through the formation of the CREB/CBP/p300 complex (Figs. 7 and 8). Interruption of either component with the respective siRNAs results in an attenuation of IL-6 expression. pCREB/CREB levels increase selectively in orbital but not in dermal fibroblasts following exposure to PGE_2_ (Fig. 4A). This phosphorylation can be blocked with a PKA inhibitor (Fig. 4B). The point of divergence between responses in dermal and orbital fibroblasts appears to reside upstream from CREB/CBP/p300 and concerns the relatively higher levels of cAMP generated in response to PGE_2_. Future studies will examine a number of other cellular responses that are more brisk in orbital cultures and might be explained by a greater capacity for these fibroblasts to generate cAMP.

IL-6 has been insinuated in the pathogenesis of several autoimmune diseases previously [7]. For instance, in synovial fibroblasts derived from patients with rheumatoid arthritis, IL-6 signaling can cross-talk with that of IL-1 [45]. IL-1 suppresses IL-6-dependent Janus kinase-STAT activity and can block the induction by IL-6 of tissue inhibitor of metalloproteinase 1. With regard to Graves' disease, elevated IL-6 levels have been described in TAO and hyperthyroidism [46]. The relatively high levels of IL-6 provoked by PGE_2_ in orbital fibroblasts may, at least in part, underlie the susceptibility of the orbit to inflammation in TAO. The cytokine could enhance lymphocyte differentiation and promote T cell trafficking to orbital tissues, an action promoted through MAPK, PI3K, and the Jak/STAT pathways [47]. IL-6 promotes the synthesis of antibodies and is necessary for the development of normal plasma cells [9]. Thus localized production of auto-antibodies could result from the high levels of IL-6 within the orbit, potentially driving their targeting of orbital antigens in TAO. It enhances monocyte differentiation into macrophages at the expense of dendritic cell development [48]. IL-4 synthesis is upregulated by IL-6 at the transcriptional level through a mechanism involving the activation of NFAT. On the other hand, IL-6 abrogates the signaling activities of interferon-γ by up-regulating suppressor of cytokine signaling-1. STAT3 activation is required for the suppression by IL-6 of LPS-dependent cell maturation. IL-6 also induces through STAT3 the *Ifi202* gene and p202 protein in mouse splenocytes [49]. These findings are proximately relevant to those reported here regarding TAO because *Ifi202* represents a candidate susceptibility gene for other autoimmune diseases such as lupus erythematosus. High-level IL-6 expression in orbital fibroblasts suggests that it might influence inflammatory responses relevant to autoimmune disease affecting the tissues surrounding the eye. Thus, PGE_2_-dependent IL-6 production in TAO might prove an important therapeutic target.

## Materials and Methods

Synthetic oligonucleotides were produced by Retrogen (Carlsbad, CA). PGE_2_, 8-bromo-Sp-cAMP, LY 294002, JNK inhibitor II, H89, and the cAMP immunoassay kit were obtained from Calbiochem/EMD Biosciences (Gibbstown, NJ). Butaprost, AH6809, and GW627368X came from Cayman Chemical (Ann Arbor, MI). Anti-phospho CREB Abs were from Millipore (Temecula, CA), and those against CREB and EP_2_ came from Cell Signaling (Boston, MA) and Abcam (Cambridge, MA), respectively. CBP siRNA was from Santa Cruz (Santa Cruz, CA) and CREB siRNA was from Dharmacon (Lafayette, CO). An ELISA kit for human IL-6 was from R & D Systems (Minneapolis, MN).

### Cell culture

Orbital fibroblast cultures were initiated as previously described [27] from tissue explants obtained during decompression surgery for severe TAO or from normal orbital tissues. Dermal fibroblasts were obtained from normal appearing skin or were purchased from the American Type Tissue Collection. These activities have been approved by the Institutional Review Board of the University of Michigan Medical Center. Fibroblasts were grown at 37°C with 95% air, 5% CO_2_ in poly-L-lysine-coated culture flasks and maintained in Dulbecco's modified Eagle's medium supplemented with 2 mM glutamine, sodium pyruvate (110 mg/ml), penicillin (100 units/ml), streptomycin (100 units/ml), 4.5% glucose and 10% fetal bovine serum (FBS). Cultures were utilized between the fifth and seventh passage from culture initiation. Medium was changed every three to four days.

### cAMP and PGE_2_ assays

cAMP levels were determined in triplicate from the cell lysates with a cAMP direct immunoassay kit (Calbiochem , San Diego, CA) following the manufacturer's protocol,. The sensitivity of the assay was 0.39 pmol/ml. Cells stimulated with adenosine 3′,5′-cyclic AMP, 8-Bromo-, and *Sp*-Isomer (Sp-cAMP), a membrane-permeable analogue served as a positive control. For PGE_2_ measurements, medium was decanted and the monolayers covered with phosphate-buffered saline in the presence of IL-1β for the final 30 min of the incubation. PBS was collected, clarified by centrifugation, and subjected to PGE_2_ EIA kit (Cayman, Ann Arbor, MI).

### Quantification of IL-6 mRNA

Total cellular RNA was isolated from cells using the RNeasy lipid tissue mini kit (Qiagen, Valencia, CA) following manufacturer's protocol. 2 units of Dnase I was treated per ∼10 µg of RNA in a 25-100 µL reaction. cDNAs were generated by reverse transcription of RNA using oligo(dT) and SuperScript III reverse transcriptase (Invitrogen Inc., Carlsbad, CA). Real-time RT-PCR was performed using cDNA preparations as templates and iQ SYBR Green Supermix (Bio-Rad, Hercules, CA) containing real-time PCR buffer, (iTaq DNA polymerase, dNTPs, SYBRGreen I, fluorescein). Primers used amplified a 645 bp DNA fragment were: forward, 5'-CAGGAGCCCAGTATAACT-3'; reverse, 5'-GAATGCCCATGCTACATTT-3' of the human IL-6 gene sequence (GenBank no. NG_011640). Quantitative RT-PCR was performed in triplicate with glyceraldehyde-3-phosphate dehydrogenase serving as the internal control on the CFX96 Real-Time PCR system (Bio-Rad). Amplification conditions consisted of initial 12-min activation at 95°C followed by 40 cycles of denaturation at 95°C for 30 s, annealing at 58°C for 30 s and extension at 72°C for 30 s. Relative quantification of the PCR amplification products was performed using the comparative critical threshold (*C*_T_) method. The Ct value from GAPDH served as an internal control for normalization.

### Transient transfections and reporter activity assays

An 1171 bp fragment, spanning –1168 to +3 nt of the human IL-6 gene promoter was described previously [25] (GenBank no. NG_011640) and cloned into pGL2-basic (Promega Inc. Madison, WI). Transient transfection of this and control constructs into fibroblasts was achieved using Effectene reagent (Qiagen, Valencia, CA) according to the manufacturer's protocol. Briefly, 2 µg of IL-6-luciferase DNA construct was transfected. Luciferase activity was measured after 48 h by the dual assay system (Promega Inc., Madison, WI). To assess transfection efficiency, 0.25 µg of pRL-TK plasmid DNA thymidine kinase promoter-driven Renilla luciferase (Promega, Madison, WI) was co-transfected with the constructs. Following cell lysis, luciferase reporter activity was assessed in 20 µl of cell extract which was mixed with 100 µl of the luciferase assay reagent, and firefly luciferase activity measured as light output (10 s) in a luminometer (Berthold Detection Systems, Huntsville, Al). The IL-6 promoter-driven luciferase enzyme activity was expressed as a ratio to the corresponding pRL-TK activity per unit of cellular protein.

To knock-down CREB, siRNA targeting human CREB and a control scrambled siRNA (-ve siRNA) obtained from Dharmacon (Lafayette, CO) at a concentration of 100 nM using RNAi as the transfection reagent (Qiagen, Valencia, CA). Following incubations, cell lysates (15 µg protein) were subjected to Western blot analysis. Transfection efficiency was monitored by Western blot analysis.

### Western blot analysis

Cellular proteins were solubilized in ice-cold harvest buffer containing 0.5% Nonidet P-40, 50 mM Tris-HCl (pH 8.0), and 10 µM PMSF following the treatments indicated. Cell lysates were taken up in Laemmli buffer and subjected to SDS-PAGE, and separated proteins were transferred to Immobilon membrane (Millipore). These were incubated with primary mAbs overnight at 4°C, washed and re-incubated with secondary peroxidase-labeled Abs. The ECL (Amersham Biosciences) chemiluminescence system was used to generate signals. Densitometric analysis of digitized images was performed with Image J software (NIH, Bethesda, Maryland) and band intensity normalized to that of the corresponding β-actin band.

### Quantification of IL-6 production

Confluent fibroblast monolayers in 24-well plates were shifted to medium without or with PGE_2_ (unless stated otherwise, at a concentration of 1 µM) or IL-1β (10 ng/ml) alone or in combination with other test compounds. In some studies, cells were serum-starved for 20 hrs and were then treated with H89 (10 µM), SB203580 (10 µM), PD98059 (10 µM), or Sp-cAMP (1 mM) for 16 hrs. Following incubations, aliquots of medium were collected and subjected to a specific ELISA for IL-6. Samples were assayed in triplicate using a standard curve.

### Flow cytometry

Techniques used in these studies have been published previously [28]. Briefly, 1×10^6^ cells were placed in 12×75-mm polypropylene tubes and fluorochrome-conjugated mAbs were added (1 µg/10^6^ cells). These were then incubated in the dark for 20 min at room temperature. Cells were washed twice with staining buffer, re-suspended in Cytofix (BD Biosciences), and kept in the dark at 4°C. Within 24 h, analysis was performed on a FACSCalibur flow cytometer (BD Biosciences). Mean fluorescent intensity (MFI) was calculated as a ratio of mean fluorescence sample/isotype fluorescence. To quantify binding sites represented by fluorescence signals, 50 µl of Quantum Simply Cellular Microbeads (Sigma-Aldrich) were incubated with 10 µl of anti-EP_2_ or isotype Ab for 30 min at room temperature. The bead standards consisted of four populations of microbeads coated with anti-rabbit Ab. Each binds a different number of mouse IgG mAb molecules (4,063, 14,354, 54,401, and 203,303 molecule-binding capacity). After Ab addition, beads were washed three times with staining buffer. Flow cytometric analysis was performed using the same settings as for cell analysis. A histogram of green fluorescence (FL1) was produced for the beads and the mean fluorescence channel number for each peak was taken. A best-fit curve was drawn to relate linear channel number to logarithmic binding capacity (molecules) from which values for the EP_2_ and isotype controls could be read. These were corrected for auto-fluorescence and nonspecific binding (QuickCal; QSC calibration software). To calibrate the fluorescence scale of the flow cytometer, we determined the Ab-binding capacity (ABC) which represents the number of Ab molecules bound on each cell or microbead.

### Co-immunoprecipitation of nuclear proteins

Nuclear proteins were isolated using the NE-PER extraction kit (Pierce Biotechnology, Rockford, IL). 100 µg of nuclear protein was pre-cleared with 40 µl of 50% Protein A-agarose slurry (1∶1 dilution) (Pierce) in lysis buffer containing 25 mM Tris (pH 8.0), 100 mM NaCl, 10% glycerol, Nonidet P-40, 1.5 mM MgCl_2_, 1 mM DTT, 1 mM PMSF, 20 µg/ml leupeptin, and 1 µg/ml pepstatin A. All procedures were carried out on ice unless stated otherwise. Samples were centrifuged and 10 µl of anti-CBP antibody was incubated overnight with gentle agitation. Fifty µl of the protein A-agarose slurry (1∶1 dilution) was added and incubated for 2 h. Protein A-agarose beads were washed in lysis buffer and then boiled in SDS sample buffer with 100 mM DTT. Proteins were separated on 4–15% SDS/PAGE gels and processed for Western blotting. Membranes were probed with 1∶1000 dilution of the appropriate antibodies.

### Nuclear pCREB binding to DNA

Nuclear proteins from dermal and Graves' orbital fibroblasts were isolated using the NE-PER extraction kit (Pierce Biotechnology, Rockford, IL). 10 µg of nuclear extracts were used in TransAM pCREB ELISA kit to assess the DNA-bindability of pCREB that are present in the nuclear extracts using protein binding CRE consensus DNA sequence and antibody targeted at pCREB. Specificity of the DNA-bindability was validated by using mutant DNA sequence. The TransAM^TM^ transcription assay kit (Active Motif, Carlsbad,CA) was used following the manufacturer's protocol.

### Data Analysis

All data are presented as mean ± S.D. Differences between two groups were determined by the Student's *t* test and significance was achieved at p<0.05.
